# Inhibition of SDF-1-induced migration of oncogene-driven myeloid leukemia by the L-RNA aptamer (Spiegelmer), NOX-A12, and potentiation of tyrosine kinase inhibition

**DOI:** 10.18632/oncotarget.22409

**Published:** 2017-11-06

**Authors:** Ellen L. Weisberg, Martin Sattler, Abdel Kareem Azab, Dirk Eulberg, Anna Kruschinski, Paul W. Manley, Richard Stone, James D. Griffin

**Affiliations:** ^1^ Department of Medical Oncology, Dana-Farber Cancer Institute, Boston, MA 02215, USA; ^2^ Novartis Institutes of Biomedical Research, CH-4002 Basel, Switzerland; ^3^ Noxxon Pharma, Max-Dohrn-Strasse 8-10, 10589 Berlin, Germany; ^4^ Department of Medicine, Harvard Medical School, Boston, MA 02115, USA

**Keywords:** chronic myeloid leukemia, SDF-1, NOX-A12, nilotinib, drug resistance

## Abstract

Resistance to targeted tyrosine kinase inhibitors (TKI) remains a challenge for the treatment of myeloid leukemias. Following treatment with TKIs, the bone marrow microenvironment has been found to harbor a small pool of surviving leukemic CD34+ progenitor cells. The long-term survival of these leukemic cells has been attributed, at least in part, to the protective effects of bone marrow stroma. We found that the NOX-A12 'Spiegelmer', an L-enantiomeric RNA oligonucleotide that inhibits SDF-1α, showed *in vitro* and *in vivo* activity against BCR-ABL- and FLT3-ITD-dependent leukemia cells. NOX-A12 was sufficient to suppress SDF-1-induced migration *in vitro*. The combination of NOX-A12 with TKIs reduced cell migration in the same *in vitro* model of SDF-1-induced chemotaxis to a greater extent than either drug alone, suggesting positive cooperativity as a result of the SDF-1 blocking function of NOX-A12 and cytotoxicity resulting from targeted oncogenic kinase inhibition. These results are consistent with our *in vivo* findings using a functional pre-clinical mouse model of chronic myeloid leukemia (CML), whereby we demonstrated the ability of NOX-A12, combined with the ABL kinase inhibitor, nilotinib, to reduce the leukemia burden in mice to a greater extent than either agent alone. Overall, the data support the idea of using SDF-1 inhibition in combination with targeted kinase inhibition to override drug resistance in oncogene-driven leukemia to significantly diminish or eradicate residual leukemic disease.

## INTRODUCTION

CML is a malignancy of hematopoietic stem cells caused by the t(9;22) chromosome translocation product BCR-ABL [1]. Excessive proliferation and abnormal trafficking of transformed progenitor cells provides a survival advantage that is accompanied by premature release of these cells into the bloodstream of patients. This has been partially attributed to BCR-ABL-altered integrin expression or function affecting the communication between leukemic cells and the bone marrow stroma, as well as with important extracellular matrix proteins such as fibronectin [2–6]. Abnormal trafficking is also marked by the homing of malignant cells to bone marrow stroma and other tissue beds characterized by having high stromal cell content. This can lead to persistent minimal residual disease in CML patients undergoing therapy with targeted inhibitors such as imatinib and nilotinib, as stromal cells secrete growth factors and protect leukemia cells from targeted inhibitors [7]. Thus, in one study, CML recurred in more than 50% of patients who discontinued imatinib therapy after having achieved and maintained at least a 5-log reduction in BCR-ABL mRNA transcript levels, or a complete molecular response [8]. In a similar study where patients discontinued nilotinib, 51.6% of patients remained in remission 48 weeks after stopping treatment [9]. Residual BCR-ABL-positive leukemic stem cells in the bone marrow of TKI-treated patients with sustained undetectable molecular residual disease may lead to relapse following termination of TKI treatment [10].

Splenic stroma is also a source of soluble cytokines associated with hematopoiesis, and splenocytes provide viability signals that lead to growth and survival of malignant cells [11–12]. Thus, in addition to bone marrow stroma, splenic stroma also can influence the viability and growth of malignant cells [13].

Early studies conducted by our group investigated the possibility that stromal cell-secreted cytokines might be able to confer cytoprotection to tyrosine kinase inhibitor-treated BCR-ABL-expressing cells. We found that a cytokine cocktail consisting of stromal cell-derived factor-1 (SDF-1 (or CXCL-12)), a bone marrow-secreted chemoattractant that supports stem cell homing, as well as a panel of cytokines secreted in high concentrations from human stromal cells, including interleukin (IL)-6, IL-8, IL-11, GM-CSF, and M-CSF [14], enhanced the growth of BCR-ABL-expressing cells and partially protected imatinib-treated leukemic cells [15]. Our findings are consistent with other published findings that demonstrated cytokine protection of chemotherapy-treated myeloid leukemia cells [16].

Clinical trial data show that the residual disease can persist in the bone marrow of patients with acute myeloid leukemia (AML) expressing oncogenic FLT3 that are treated with the small molecule inhibitor, midostaurin [17–20]. These findings suggest that, as with what has been observed for kinase inhibitor-treated CML, stromal cells similarly provide viability signals to AML cells that protect them from the effects of kinase inhibition.

One approach to potentially override the cytoprotective effect of stroma on TKI-treated leukemic cells involves targeting the CXCR4 chemokine receptor and its ligand, SDF-1, which constitute a chemokine/receptor axis that mediates hematopoietic cell migration to bone marrow stroma. CXCR4 antagonists direct leukemic cells away from cytoprotective stroma and thus have the potential to enhance the apoptosis-inducing effects of TKIs like imatinib [21]. Plerixafor (AMD3100; Mozobil™; Genzyme), an antagonist/partial agonist of CXCR4 and allosteric agonist of CXCR7 [22], is used clinically for harvesting hematopoietic stem cells for use in autologous transplantation in patients with non-Hodgkin's lymphoma and multiple myeloma (MM). We and others have shown that plerixafor potentiates the cytotoxic effects of chemotherapy- or TKIs- on stroma-protected AML [23–25], MM [26–28], and CML [29–31]. Clinical trials investigating plerixafor and other agents targeting CXCR4, including anti-CXCR4 monoclonal antibodies and peptide CXCR4 antagonists, for leukemia and lymphoma are currently under way [32].

An alternative to inhibiting the CXCR4 receptor is targeting its ligand, SDF-1, directly. SDF-1 is secreted and presented by stromal cells from bone marrow and other tissues by cell-surface-bound glycosaminoglycans (GAGs); this attracts leukemia cells and contributes to residual disease by conferring protection to the cytotoxic effect of small molecule inhibitors. NOX-A12 is an RNA oligonucleotide in L-configuration (or the mirror image of naturally occurring RNA) (Spiegelmer), which makes it unrecognizable by plasma nucleases and resistant to degradation, and which was originally designed to interfere with SDF-1 in the stromal microenvironment [33–34]. NOX-A12 has been shown to inhibit SDF-1-induced chemotaxis of chronic lymphocytic leukemia (CLL) cells and conversely augment migration of CLL cells beneath a bone marrow stromal cell (BMSC) layer by disrupting the BMSC-generated SDF-1 gradient [35]. It has also been found to reduce MM tumor burden in mice when combined with bortezomib [36], as well as to exhibit activity against glioblastoma in rat models [37] and potentiate anti-VEGF therapy against glioblastoma [38]. NOX-A12 was tested in healthy volunteers and found to be well-tolerated and to dose-dependently mobilize white blood cells and hematopoietic stem cells into the peripheral blood [39]. Thus far, encouraging results have been obtained in clinical trials testing NOX-A12 in combination with bendamustine and rituximab (BR) for the treatment of relapsed/refractory CLL patients, and testing of NOX-A12 in combination with bortezomib (Velcade) and dexamethasone (VD) in relapsed/refractory MM patients [40].

To date, to the best of our knowledge, therapeutic effects of NOX-A12 have not been explored in myeloid leukemia. Here, we show the ability of NOX-A12 to block SDF-1-induced migration of BCR-ABL-positive leukemia cells, as well as *FLT3-ITD*-positive leukemia cells. In addition, we demonstrate the combined effects of targeted kinase inhibition and NOX-A12 *in vitro* and *in vivo*.

## RESULTS

### NOX-A12 inhibits SDF-1-induced migration of BCR-ABL-expressing cells and potentiates the effects of ABL inhibition against BCR-ABL-positive cells *in vitro*

**Figure 1:**
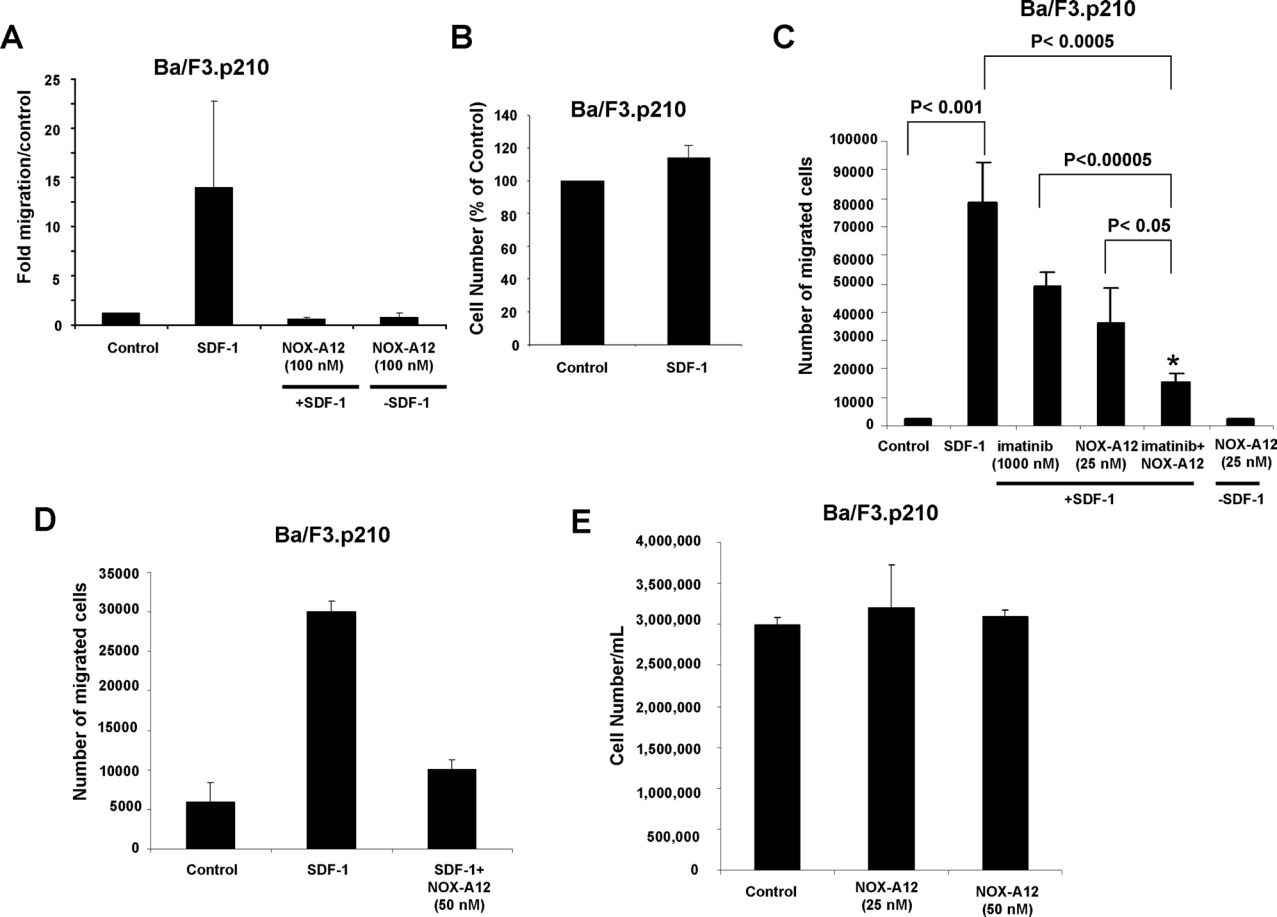
NOX-A12 inhibition of SDF-1-induced migration of BCR-ABL-expressing Ba/F3 cells and potentiation of effects of ABL inhibition against BCR-ABL-positive Ba/F3 cells *in vitro* (**A**) Transwell migration assay (*n* = 1); error bars (standard deviation) represent intra-experimental variability, with multiple quantifications taken for one Transwell migration assay. Ba/F3.p210 cells were stimulated with mSDF-1 (100 ng/mL) in the absence or presence of NOX-A12 (100 nM). Data are presented as fold migration/control, where control is normalized to a value of 1. Transwell migration assay incubation time was overnight for Ba/F3.p210 cells. Data shown are mean +/− S.D. (**B**) Effect of mSDF-1 (100 ng/mL) on proliferation of Ba/F3.p210 cells following approximately 2 days of treatment. (**C**) Transwell migration assay: Ba/F3.p210 cells stimulated with mSDF-1 (100 ng/mL) in the presence of 25 nM NOX-A12, 1000 nM imatinib, or a combination of both. Transwell migration assay incubation time was overnight to 24 hours. Results shown are a composite of 3–4 independent experiments and error bars (standard deviation) represent inter-experimental variability. Data shown are mean +/− S.D. 2-sided *t*-test, *p* values: Control versus SDF-1 is statistically significant (*p* = 0.00076). ^*^SDF-1 versus SDF-1+imatinib+NoxA12 is statistically significant (*p* = 0.00032). ^*^SDF-1+imatinib versus SDF-1+imatinib+NOX-A12 is statistically significant (*p* = 0.00003). ^*^SDF-1+NOX-A12 versus SDF-1+imatinib+NOX-A12 is statistically significant (*p* = 0.01758). Control versus NOX-A12 in the absence of SDF-1 is not statistically significant (*p* = 0.79296). (**D**) Transwell migration assay (*n* = 1); error bars (standard deviation) represent intra-experimental variability, with multiple quantifications taken for one Transwell migration assay. Ba/F3.p210 cells were stimulated with mSDF-1 (100 ng/mL) in the absence or presence of NOX-A12 (50 nM). Data are presented as number of migrated cells. Transwell migration assay incubation time was 24 hr. Data shown are mean +/− S.D. (**E**) Effect of NOX-A12 (25–50 nM) on proliferation of Ba/F3.p210 cells following approximately 24 hr of treatment. Data are presented as cell concentration (cell number/mL). Data shown are mean +/− S.D.

In transwell migration assays designed to test the ability of NOX-A12 to inhibit SDF-1-induced migration of BCR-ABL-expressing cells, we found that 50–100 nM NOX-A12 effectively inhibited the SDF-1-stimulated migration of BCR-ABL-expressing Ba/F3 and 32D cells and reduced migration to a level similar to unstimulated control cells (Figure 1A, 1D and Supplementary Figure 1A). Specifically, SDF-1 induced migration of Ba/F3.p210 cells by 5–11.2 fold over control (Figure 1A, 1D), whereas in the presence of NOX-A12 (50–100 nM), SDF-1 only induced migration by 0.5–0.6 fold (Figure 1A, 1D). Similarly, SDF-1 induced migration of 32D.p210 cells by 23.1 fold, whereas in the presence of NOX-A12, SDF-1 only induced migration by 2 fold (Supplementary Figure 1A). Effects of SDF-1 observed in the transwell migration assay were confirmed to be due to effects on migration as opposed to increased growth of cells, as the proliferation of Ba/F3.p210 cells treated with SDF-1 for two days was found to be unchanged (Figure 1B). Specifically, in contrast to the 11.2-fold induction of Ba/F3.p210 cell migration by SDF-1 (Figure 1A), we observed a mere 1.1-fold difference between untreated and SDF-1-stimulated Ba/F3.p210 cells with respect to proliferation. In addition, we showed in a separate set of studies performed in parallel that 19 hours of stimulation of Ba/F3.p210 cells with SDF-1 led to a strong induction of cell migration in a transwell migration assay, whereas 19 hours of SDF-1 stimulation of Ba/F3.p210 cells did not lead to any measurable increase in cell proliferation (Supplementary Figure 1B–1C). We also tested (in parallel) the effects of NOX-A12 (50 nM) on proliferation as compared to migration, and we observed that while NOX-A12 strongly suppressed SDF-1-induced migration, it did not enhance or inhibit cell proliferation (Figure 1D–1E). These data are in support of the effects of NOX-A12 observed in the transwell migration assay being due to effects on migration as opposed to increased cell growth.

Importantly, NOX-A12 treatment combined with imatinib treatment of BCR-ABL-expressing cells resulted in reduced SDF-1-induced migration of cells as compared to NOX-A12 or imatinib alone, suggesting a positive combination effect of the two inhibitors in this *in vitro* assay when used together, likely due to direct inhibitory effects of NOX-A12 on SDF-1-induced cell migration coupled with cell cytotoxicity resulting from BCR-ABL kinase inhibition (Figure 1C). The difference between migration of control cells versus cells stimulated with SDF-1 was statistically significant (*p* < 0.001), and the differences between imatinib alone or NOX-A12 alone (each in the presence of SDF-1) and the combination of imatinib and NOX-A12 (in the presence of SDF-1) were statistically significant (*p* < 0.00005 and *p* < 0.05, respectively). The difference between migration for cells in the SDF-1 treatment group and migration for cells treated with imatinib+NOX-A12 in the presence of SDF-1 was significant (*p* < 0.0005), and there was no statistically significant difference between migration of control cells and cells treated with NOX-A12 in the absence of SDF-1 (*p* = 0.79).

**Figure 2:**
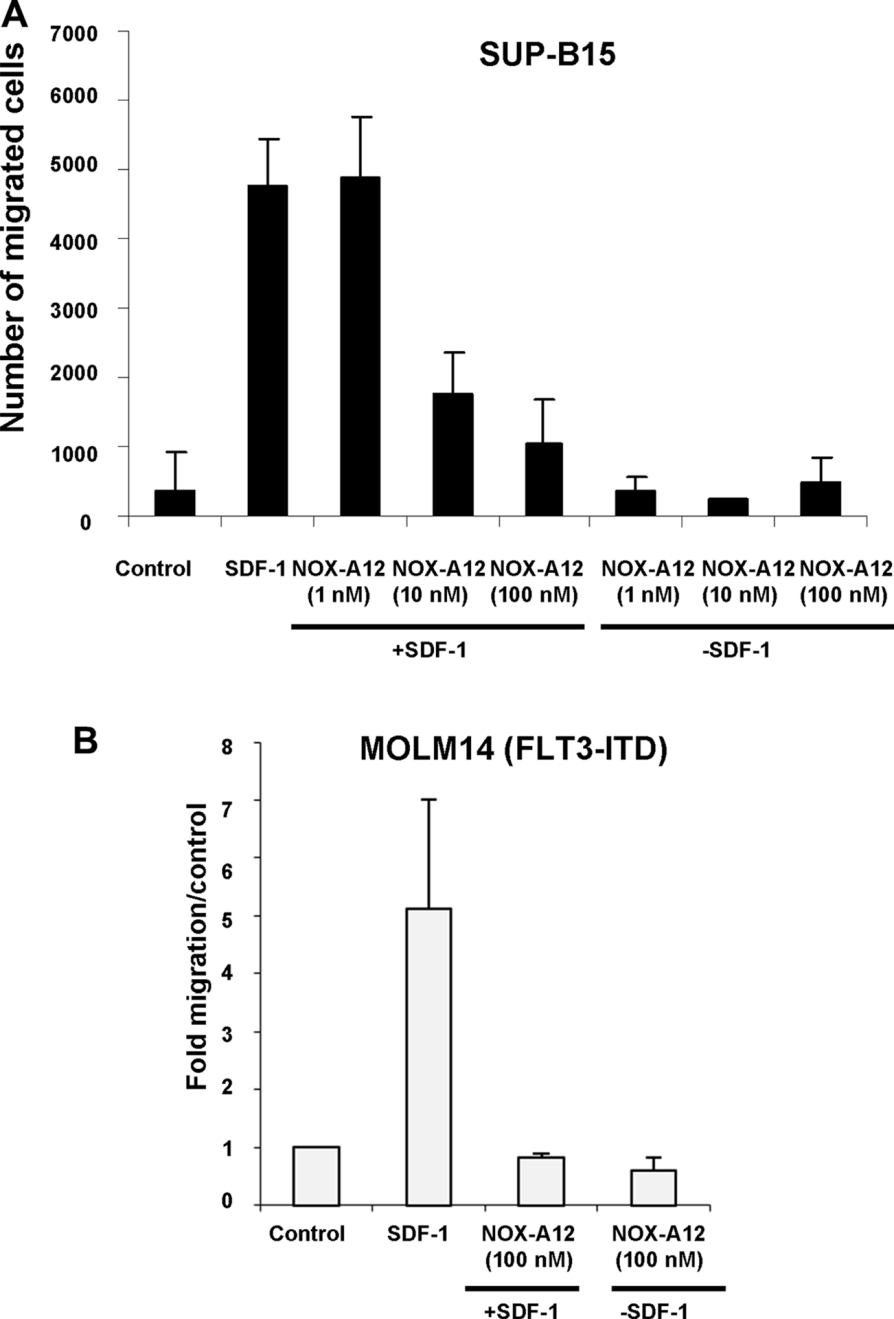
NOX-A12 inhibition of SDF-1-induced migration of BCR-ABL-expressing human SUP-B15 cells and FLT3-ITD-positive human MOLM14 cells (**A**) Transwell migration assay (*n* = 1); error bars (standard deviation) represent intra-experimental variability, with multiple quantifications taken for one Transwell migration assay. SUP-B15 cells were stimulated with hSDF-1 (100 ng/mL) in the presence of NOX-A12 (1–100 nM). Data are presented as number of migrated cells. Transwell migration assay incubation time was 24 hours for SUP-B15. Data shown are mean +/− S.D. (**B**) Transwell migration assay (*n* = 1); error bars (standard deviation) represent intra-experimental variability, with multiple quantifications taken for one Transwell migration assay. MOLM14 cells were stimulated with hSDF-1 (50 ng/mL) in the presence of NOX-A12 (100 nM). Data are presented as fold migration/control, where control is normalized to a value of 1. Transwell migration assay incubation time was overnight. Data shown are mean +/− S.D.

The anti-SDF-1 effect of NOX-A12 was not limited to murine cells, as evidenced by its ability to reduce, however not abolish, SDF-1-induced migration of the human BCR-ABL-positive acute lymphoblastic leukemia (ALL) line, SUP-B15, and the human *FLT3-ITD*-positive line, MOLM14 (Figure 2). SUP-B15 cells, although not a myeloid line, is being used in our study as a representative human cell line model of Philadelphia chromosome-positive leukemia that is dependent on the BCR-ABL oncogene. Specifically, SDF-1 stimulation of SUP-B15 cells led to a 12.7-fold induction of migration of cells, whereas SDF-1 stimulation of SUP-B15 cells in the presence of NOX-A12 (10 nM) led to only a 4.7-fold increase in cell migration, and in the presence of NOX-A12 (100 nM) led to only a 2.8-fold increase in cell migration (Figure 2A).

### NOX-A12 inhibits SDF-1-induced migration of FLT3-ITD-expressing cells and potentiates the effects of FLT3 inhibition against mutant FLT3-positive cells

As stromal cell-mediated chemoresistance has proven to be a challenge for TKI-treated leukemia, and TKIs, such as midostaurin, are now in clinical use for mutant FLT3-positive leukemia, it was of interest to explore the clinical and cooperative potential of NOX-A12 in the context of this disease. We first found that SDF-1 stimulation of MOLM14 cells led to a 4.96-fold induction of migration of cells, whereas SDF-1 stimulation of MOLM14 cells in the presence of NOX-A12 (100 nM) led to a 0.82-fold induction of migration of cells (Figure 2B). We next observed that NOX-A12 effectively inhibited the SDF-1-stimulated migration of *FLT3-ITD*-expressing MOLM13-luc+ cells in a transwell migration assay and reduced migration to a level similar to unstimulated control cells (Supplementary Figure 2A). Specifically, SDF-1 stimulation led to migration of 5,125 cells, as compared to 1,375 migrated cells in the control, whereas SDF-1 stimulation in the presence of NOX-A12 (25 nM) led to migration of only 1,500 cells (Supplementary Figure 2A). In addition, the combination of NOX-A12 (at 25 nM) and midostaurin led to slightly reduced SDF-1-induced migration of MOLM13-luc+ (813 cells) as compared to NOX-A12 alone (1,500 cells) or midostaurin alone (2,188 cells) in the transwell migration assay (Supplementary Figure 2A). A similar combination study was carried out for MOLM14 cells (Figure 2B). As expected, we observed robust SDF-1 induction of cell migration, which was strongly suppressed by 25 nM NOX-A12. The combination of midostaurin and NOX-A12 (at 25 nM) similarly led to slightly greater suppression of MOLM14 cell migration as compared to single agent-treated cells (Supplementary Figure 2B).

### NOX-A12 potentiates the anti-proliferative effects of ABL inhibition against BCR-ABL-positive cells *in vivo*

The simultaneous demonstrated anti-SDF-1 influence of NOX-A12 coupled with cell killing by TKI encouraged further studies exploring the ability of NOX-A12 to potentiate the effects of ABL inhibition *in vivo*, presumably by mobilizing BCR-ABL-positive leukemia cells out of bone marrow and into peripheral blood due to direct inhibition of SDF-1 and consequent blockade of its function in the stromal microenvironment as a chemoattractant. We utilized a mouse model of BCR-ABL-positive leukemia to examine the *in vivo* effects of SDF-1 inhibition by NOX-A12 (40 mg/kg every other day administered subcutaneously (sc)), combined with the ABL inhibitor, nilotinib, administered by oral gavage. In a pilot study designed to determine the optimal dose of nilotinib to use in combination with NOX-A12, mice treated for four days with 30mg/kg nilotinib showed strong and rapid loss of leukemia burden, whereas NOX-A12 alone did not inhibit *in vivo* leukemia growth (data not shown). As the effects of nilotinib were disproportionate to the effects of NOX-A12, the nilotinib dose was reduced by 2-fold and mice were treated orally for a total of 11 days with 15mg/kg nilotinib in the presence or absence of 40 mg/kg NOX-A12 every other day sc. Nilotinib-treated mice were observed to carry very low leukemia burden following 15 days total treatment (Supplementary Figure 3A, 3B) as compared to vehicle-treated mice and NOX-A12 only-treated mice. However, the combination of NOX-A12 and nilotinib led to further suppression of leukemia growth in 2 of 3 mice in the combination group as compared to nilotinib only-treated mice (Supplementary Figure 3C).

**Figure 3:**
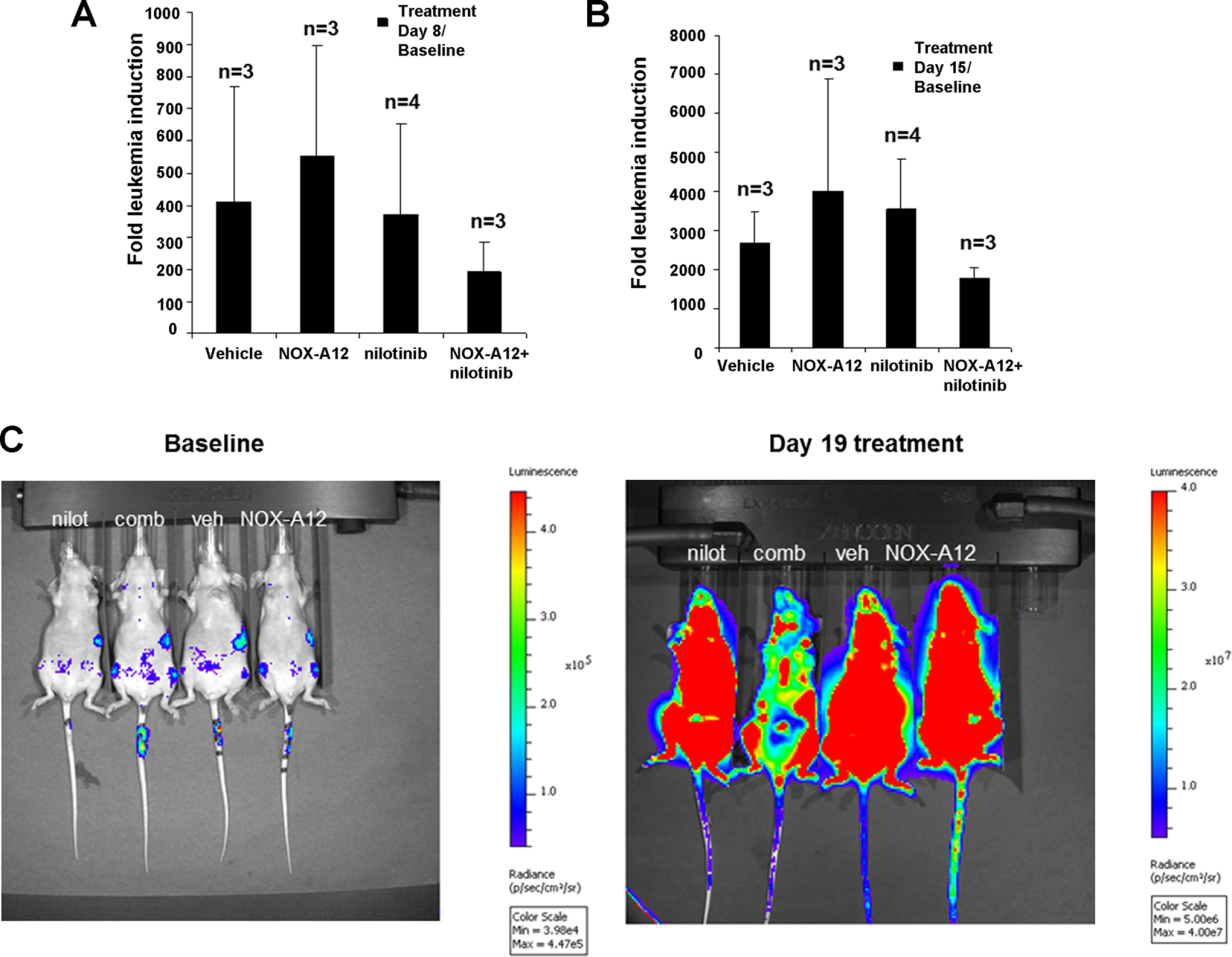
NOX-A12 potentiation of effects of ABL inhibition against BCR-ABL-positive cells *in vivo*: Effects on total body leukemia burden Mice were IV-injected via tail vein with 1 million 32D.p210-luc+ cells and imaged 3 days later to establish baseline bioluminescence. Mice were randomized into the following four treatment groups: Vehicles (administered NMP/PEG solution via oral gavage every day), NOX-A12-only (40 mg/kg sc every other day), nilotinib (7.5mg/kg oral gavage every day), or a combination. Mice were continuously administered treatments until morbidity. Mice were preserved in 10% formalin for histopathological analysis. (**A**–**B**) Fold leukemia induction (32D.p210-luc+ cells IV injected via tail vein) for treatment groups on treatment day 8 (A) and treatment day 15 (B); “n” refers to the number of mice in each treatment group. Bioluminscence values for one NOX-A12-only mouse and one combination-only mouse were too low to be considered reliable on day 15 of treatment and were therefore not included in determination of fold leukemia induction for treatment day 15 and treatment day 8 (for consistency). (**C**) Shown here are bioluminescent images for four representative mice prior to treatment (baseline) and on day 19 of treatment.

**Figure 4:**
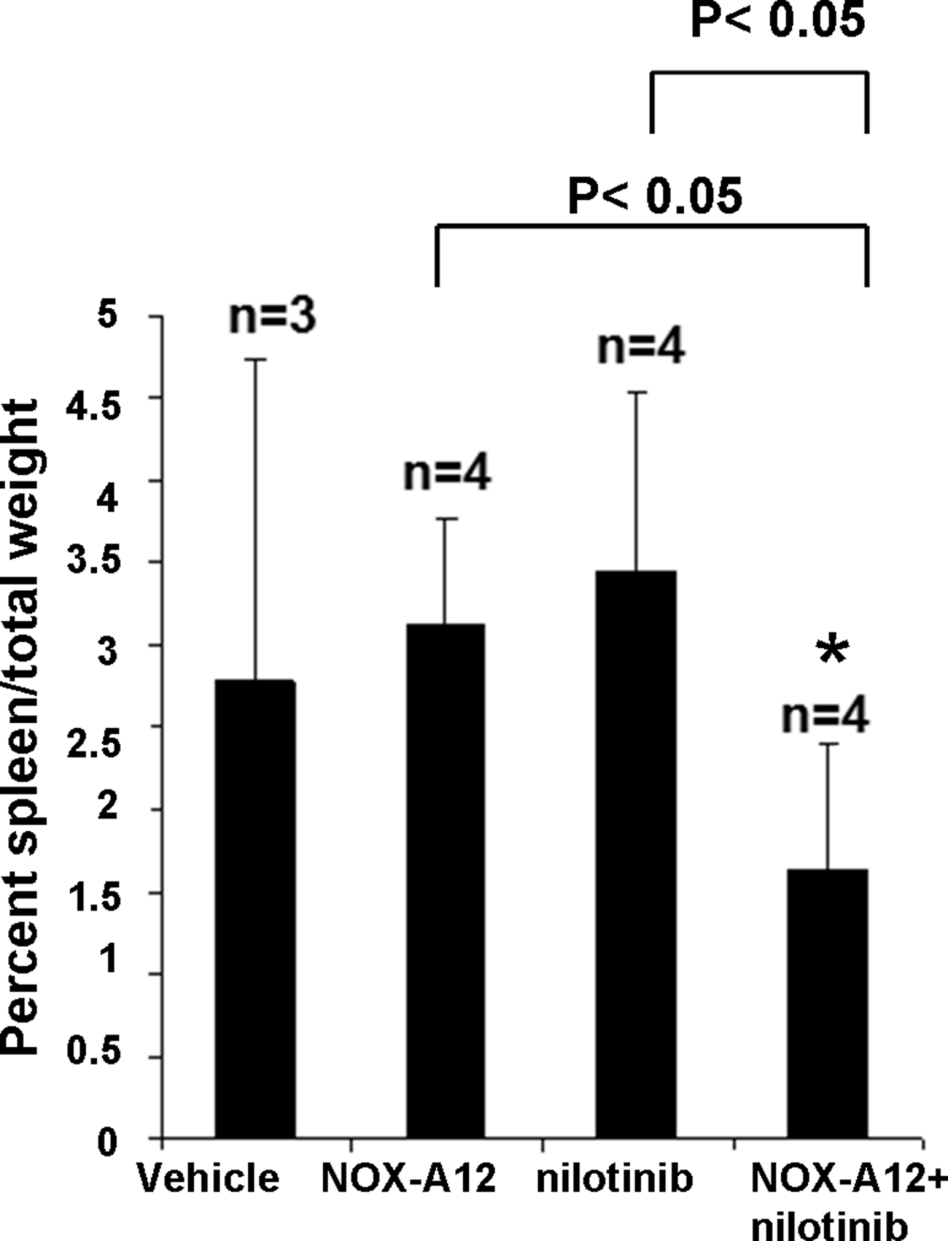
NOX-A12 potentiation of effects of ABL inhibition against BCR-ABL-positive cells *in vivo*: Effects on spleen weight Percent spleen/total weights for treatment groups. ^*^NOX-A12 versus combination statistically significant (*p* = 0.025643). ^*^Nilotinib versus combination statistically significant (*p* = 0.036021).

The results of the pilot study encouraged us to test nilotinib at a lower dose (7.5 mg/kg every day administered by oral gavage) alone and in combination with NOX-A12 in BCR-ABL-positive leukemia-bearing mice. As expected, NOX-A12 showed no single-agent activity, however in combination with nilotinib it led to reduced total leukemia burden assessed by total body bioluminescence (Figure 3A–3C). Spleens dissected from vehicle- and drug-treated mice were significantly smaller in the drug combination group than in vehicle-treated or single agent-treated mice (Figure 4). The size of spleens of NOX-A12-treated mice were significantly different from the spleen sizes of combination-treated mice (*p* = 0.02564), and the size of spleens of nilotinib-treated mice were significantly different from the spleen sizes of combination mice (*p* = 0.03602). Drugs, alone and combined, were generally well-tolerated by mice with no adverse toxicity to vital organs (data not shown). Since NOX-A12 exerted no efficacy as a single agent, these results demonstrate that NOX-A12, at a well-tolerated dose, acts cooperatively with nilotinib to suppress the growth of 32D.p210 leukemia.

## DISCUSSION

The important role of stroma in leukemia cell trafficking is evident from the number of studies that have been carried out over the past couple of decades. Abnormal adhesion properties are believed to interfere with stroma:leukemia cell interaction and lead to increased leukemic cell proliferation and the untimely release of these cells out of patient bone marrow and into the peripheral blood. The converse homing of leukemic cells to bone marrow stroma dampens the efficacy of TKI therapy because of cytoprotection of leukemic cells and consequent development of minimal residual disease. New approaches to overriding stromal cell-associated chemoresistance, such as the use of agents that inhibit the CXCR4-SDF-1 axis, could potentially improve leukemia patient response and survival if developed as anticancer therapeutics. Of relevance in the case of CML, while there is a reduction of CXCR4 expression and signaling in BCR-ABL-positive cells, conversely treatment with imatinib or nilotinib increases CXCR4 surface expression on CML cells; this causes these cells to migrate to cytoprotective bone marrow stroma, rendering them resistant to TKI therapy [29, 41, 42].

NOX-A12 is an RNA oligonucleotide in L-configuration (Spiegelmer^®^) that binds and neutralizes SDF-1 thereby blocking its interaction with CXCR4 and CXCR7 and which also disrupts existing SDF-1 gradients by stripping the chemokine from GAGS [35]. By inhibiting SDF-1 with NOX-A12, the tumor microenvironment can be modulated in such a way as to mobilize leukemia cells to the peripheral blood where they are more susceptible to the cytotoxicity of anti-cancer agents. Additionally, as SDF-1 behaves as a survival factor for leukemic cells, blockade of SDF-1 could potentially reduce leukemia cell viability [40]. NOX-A12, because of its ability to block SDF-1 activity on both of its receptors, CXCR4 and CXCR7, provides additional benefit over a targeted CXCR4 receptor inhibitor because of its dual influence on CXCR7, which affects proliferation, migration, and metastasis of some cancers [43].

To the best of our knowledge, the activity of NOX-A12 alone and in combination with targeted therapies has not been explored for CML and AML. Here, we observed the ability of NOX-A12 to potentiate the effects of ABL inhibition against BCR-ABL-positive leukemia *in vitro* and *in vivo*. In transwell migration assays, NOX-A12 inhibited SDF-1-induced migration of BCR-ABL-expressing Ba/F3, 32D, and SUP-B15 (human Ph+ ALL) cells. NOX-A12 also potently inhibited SDF-1-induced migration of *FLT3-ITD*-expressing MOLM13 and MOLM14 cells.

Using a murine model of BCR-ABL-positive progressive disease, we found that NOX-A12 combined with nilotinib was more effective in reducing leukemia burden than either drug alone. In our models, NOX-A12 modestly enhanced the efficacy of nilotinib when nilotinib, as a single agent, was used at doses that led to substantial suppression of leukemia. However, a stronger positive combination effect was observed between NOX-A12 and a lower dose of nilotinib that was ineffective when administered as monotherapy. As such, combination treatment may have a particularly effective role in those patients not able to tolerate TKI treatment and for whom dose reduction of the TKI is needed.

Taken together, our data confirm the anti-SDF-1 activity of the ‘Spiegelmer’ and L-enantiomeric RNA oligonucleotide, NOX-A12. Our findings also suggest that NOX-A12 has the ability to potentiate the efficacy of TKIs against a subset of oncogene-driven leukemias.

## MATERIALS AND METHODS

### Cell lines and cell culture

BCR-ABL tyrosine kinase-dependent murine 32D.p210 cells were developed as described [44]. SUP-B15 cells were purchased from the American Type Culture Collection (ATCC) (Manassas, VA, USA). Ba/F3.p210 cells were obtained by transfecting the IL-3-dependent murine hematopoietic Ba/F3 cell line with a pGD vector containing p210BCR-ABL (B2A2) cDNA [45].

The human AML-derived, *FLT3-ITD*-expressing line, MOLM14, was provided to us by Dr. Scott Armstrong, Dana-Farber Cancer Institute (DFCI), Boston, MA. The human AML-derived, *FLT3-ITD*-expressing cell line, MOLM-13 (DSMZ (German Resource Centre for Biological Material), was engineered to express luciferase fused to neomycin phosphotransferase (pMMP-LucNeo) by transduction with a VSVG-pseudotyped retrovirus as previously described [46].

All cell lines used in this study were cultured with 5% CO_2_ at 37°C, at a concentration of 2 × 10^5^ to 5 × 10^5^ in RPMI (Corning, Inc. Corning, NY) with 10% HyClone fetal bovine serum (FBS) (GE Healthcare Life Sciences, Pittsburgh, PA), and supplemented with 2% L-glutamine and 1% penicillin/streptomycin.

Human cell lines were authenticated through cell line short tandem repeat (STR) profiling (DDC Medical, Fairfield, OH and Molecular Diagnostics Laboratory, Dana-Farber Cancer Institute). All cell lines tested matched > 80% with lines listed in the ATCC or DSMZ Cell Line Bank STR. SUP-B15 cells were newly purchased from ATCC at the time of this study. All cell lines were confirmed to be virus- and *mycoplasma*-free.

### Chemicals

Midostaurin (Rydapt) and nilotinib (Tasigna) were synthesized by Novartis Pharma AG, Basel, Switzerland and dissolved in DMSO to obtain a 10 mM stock solution. Serial dilutions were then made, to obtain final dilutions for cellular assays with a final concentration of DMSO not exceeding 0.1%. Stock solutions of the RNA nucleic acid, NOX-A12/olaptesed pegol (Noxxon, Germany), were prepared in 5% glucose in water at a 5 mg/mL concentration and stored as aliquots, light-protected, at -20°C.

### Proliferation studies

The trypan blue exclusion assay has been previously described [47] and was used for quantification of cells prior to seeding for CellTiter-Glo Luminescent Cell Viability assays (Promega, Madison, WI). These assays were used for cell growth studies and carried out according to manufacturer's instructions. Cell viability is reported as percentage of control (untreated) cells, and error bars represent the standard deviation for each data point.

### Transwell migration assay

For murine Ba/F3.p210 and 32D.p210 cells, the lower chamber of a Transwell plate (6.5mm Transwell® with 5.0μm Pore Polycarbonate Membrane Insert, Sterile (Product #3421), Corning Costar Corporation, Cambridge, MA) was filled with 600 μL of migration (starvation) media (RPMI 1640/PBS+0.01% bovine serum albumin (BSA)), followed by placement of inserts into the wells. For human SUP-B15, MOLM13 and MOLM14 cells, the lower chamber of a Transwell plate (6.5mm Transwell® with 8.0μm Pore Polycarbonate Membrane Insert, Sterile (Product #3422), Corning Costar Corporation, Cambridge, MA) was filled with 600 μL of migration (starvation) media (IMDM/PBS+0.05% BSA) followed by placement of inserts into the wells. Cells were counted by Trypan Blue exclusion with a hemocytometer, and resuspended in migration media at a concentration of 2 × 10^6^ cells/mL. 100 μL of this cell suspension was transferred to the upper chamber (for a total of 2 × 10^5^ cells). The media in the lower chamber contained either no SDF-1 (for control samples) or 100 ng/mL mouse SDF-1 (for Ba/F3.p210 and 32D.p210 cells) or 50–100 ng/mL human SDF-1 (for MOLM13, MOLM14, or SUP-B15 cells). NOX-A12 and kinase inhibitors were included in the assay and added to the top and bottom chambers to avoid gradient formation at the indicated concentrations. After the indicated times, cells in the lower compartment were resuspended and counted using Trypan Blue exclusion. The spontaneous Transwell migration of cells was expressed as the total number of cells that migrated in the presence of SDF-1 versus SDF-1+NOX-A12. Shown are mean+/− S.D. for each sample.

### Bioluminescent BCR-ABL model of CML

32D.p210 cells were transduced with a retrovirus encoding firefly luciferase (MSCV-Luc), and selected with G418 at a concentration of 1 mg/mL to produce the 32D.p210-luciferase (luc+) cell-line. Bioluminescence imaging was carried out as previously described [48]. Briefly, virus- and *Mycoplasma*-free cells were washed in Hank's Balanced Salt Solution (HBSS; Mediatech, Inc.,VA), resuspended in PBS for injection, and administered via IV tail vein injection (250 μL, 1 × 10^6^ cells) into 15 female NOD.Cg-*Prkdc^scid^ Il2rg^tm1Wjl^*/SzJ mice (or NOD *scid* gamma (NSG™)) (6 weeks of age) (cat # 005557), purchased from The Jackson Laboratory (Bar Harbor, ME). Mice were imaged 3 days later to establish baseline bioluminescence and were randomized into the following four treatment groups: Vehicles (administered NMP/PEG solution), NOX-A12-only (40 mg/kg sc every other day), nilotinib (7.5 mg/kg oral gavage every day), or a combination. Mice were continuously administered treatments until morbidity, at which time spleens were dissected and weighed and reported as a percentage of total mouse weight.

### Drug formulations for *in vivo* studies

Solutions of nilotinib (Novartis Pharma AG, Basel) were prepared just prior to administration, by dissolving 100 mg in 1.0 mL of NMP to give a clear solution and diluting with 9.0 mL PEG300. Working solutions of NOX-A12 were prepared by adding ca. 50% of target volume (e.g. 5 mL to 100 mg container) of 5% glucose in water and dissolving the lyophilisate under aseptic conditions. Incubation for at least 30 minutes at 37°C was required to completely dissolve the lyophilisate with occasional vortexing. The solution was rinsed two times with ca. 20% of the target volume of solution medium (e.g. 2 mL) and the rinsed solution was transferred to a preparation container. Solution medium was added to target volume (e.g. 10 mL). Aliquots prepared at 10 mg/mL were stored, light protected, as stock solutions at −20°C and thawed prior to use.

### Statistical analysis

Results were reported as the mean ± standard deviation. Samples were compared by the Student's *t*-test, and results were considered significantly different for *p* values less than 0.05.

## SUPPLEMENTARY MATERIALS FIGURES



## Abbreviations

TKI: tyrosine kinase inhibitors
CML: chronic myeloid leukemia
SDF-1 (or CXCL-12): stromal cell-derived factor-1
hSDF-1: human SDF-1
mSDF-1: mouse SDF-1
AML: acute myeloid leukemia
MM: multiple myeloma
GAGs: glycosaminoglycans
CLL: chronic lymphocytic leukemia
ALL: acute lymphoblastic leukemia
BMSC: bone marrow stromal cell
BR: bendamustine and rituximab
VD: bortezomib (Velcade) and dexamethasone
pMMP-LucNeo: luciferase fused to neomycin phosphotransferase
FBS: fetal bovine serum
BSA: bovine serum albumin
STR: short tandem repeat
ATCC: American Type Culture Collection
MSCV-Luc: retrovirus encoding firefly luciferase
luc+: luciferase
HBSS: Hank's Balanced Salt Solution
sc: subcutaneously
