# Supplementary material for: Inhibition of SDF-1-induced migration of oncogene-driven myeloid leukemia by the L-RNA aptamer (Spiegelmer), NOX-A12, and potentiation of tyrosine kinase inhibition
Source: Oncotarget. 2017 Nov 6;8(66):109973–84. doi: 10.18632/oncotarget.22409 (PMC5746358; doi:10.18632/oncotarget.22409)
Supplement: Supplementary file 1 [file oncotarget-08-109973-s001.pdf]

Inhibition of SDF-1-induced migration of oncogene-driven myeloid leukemia by the L-RNA aptamer (Spiegelmer), NOX-A12, and potentiation of tyrosine kinase inhibition

SUPPLEMENTARY MATERIALS

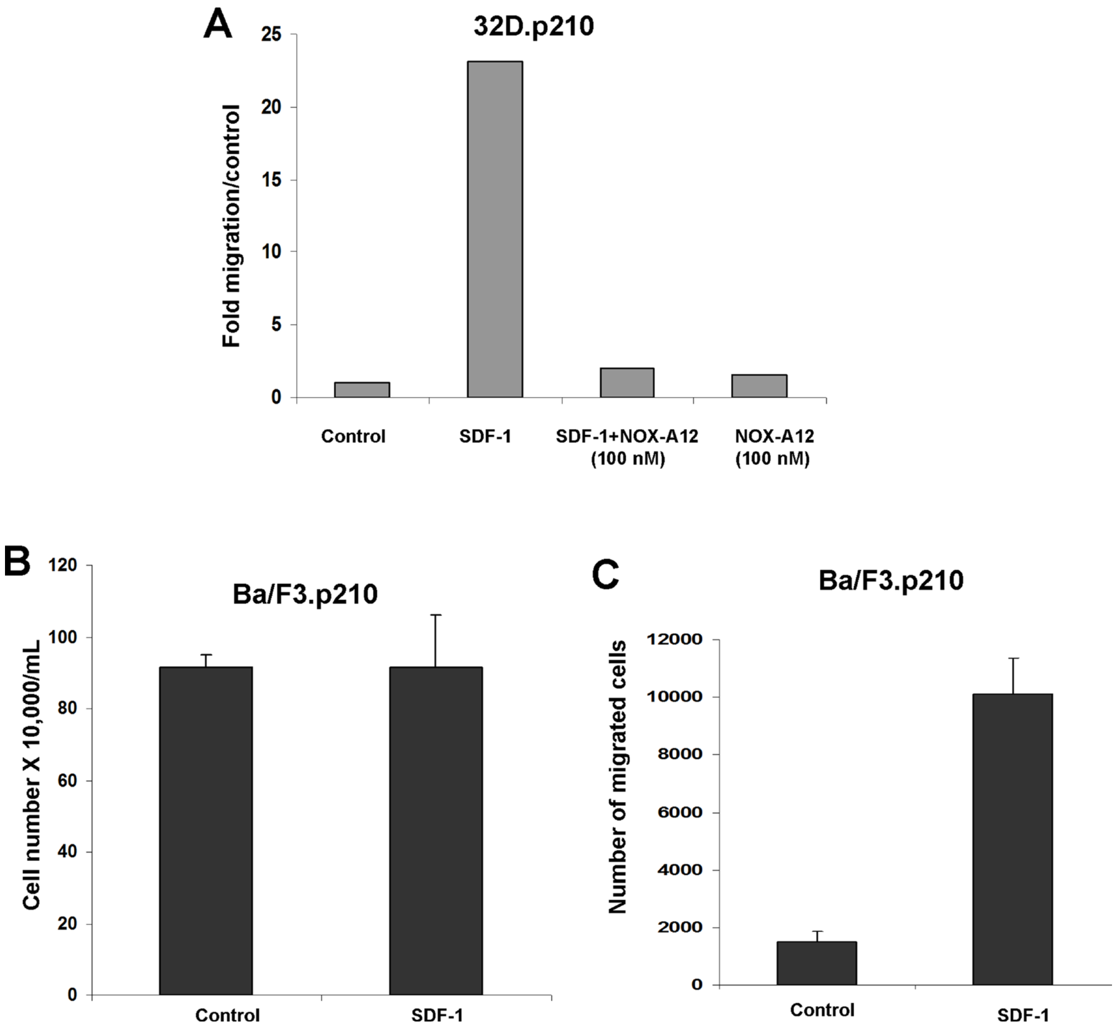

Supplementary Figure 1: Nox-A12 inhibition of SDF-1-induced migration of BCR-ABL-expressing cells. (**A**) Transwell migration assay: 32D.p210 cells stimulated with mSDF-1 (100 ng/mL). Data are presented as fold migration/control, where control is normalized to a value of 1. Transwell assay migration time was 2 days for 32D.p210 cells. (**B–C**) Approximately 19 hr proliferation assay (B) and 19 hr transwell migration assay (C): Comparison of effects of SDF-1 on Ba/F3.p210 proliferation versus migration.

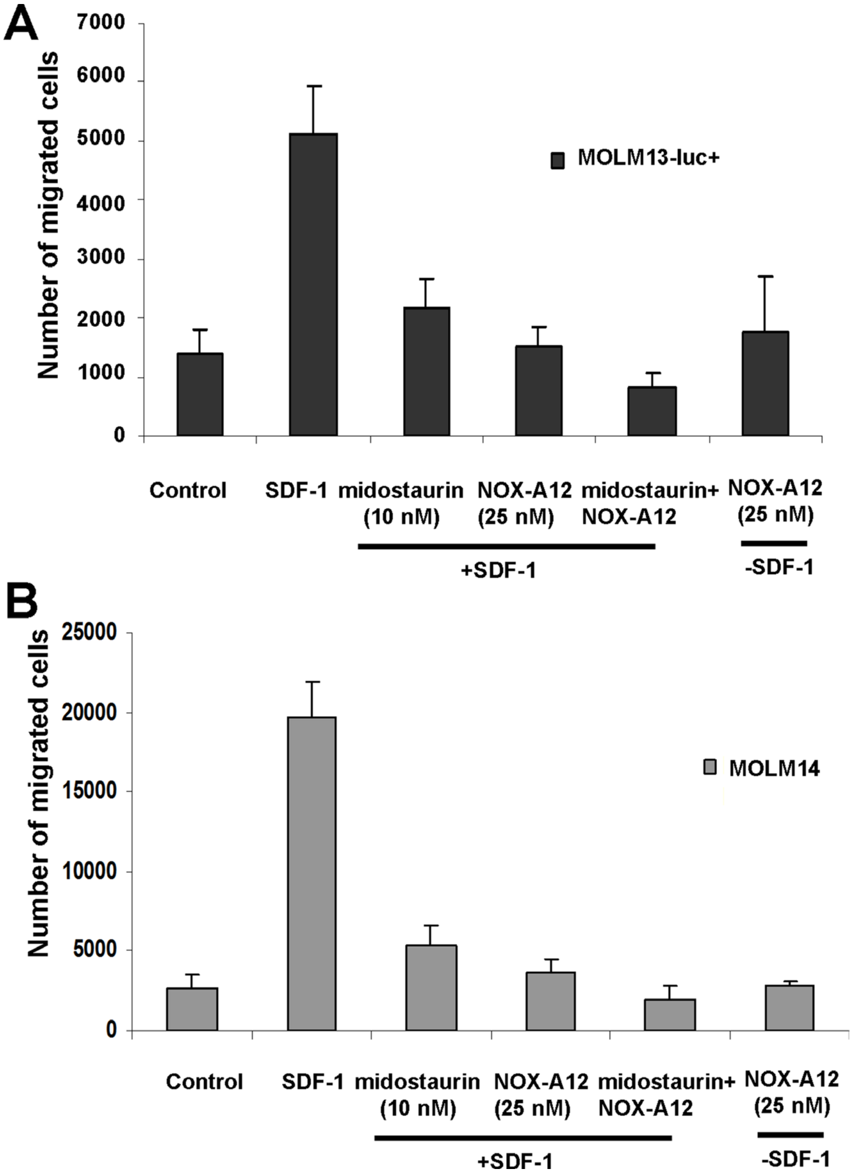

Supplementary Figure 2: NOX-A12 inhibition of SDF-1-induced migration of FLT3-ITD-expressing cells and potentiation of effects of FLT3 inhibition against mutant FLT3-positive cells *in vitro*. (**A**–**B**) Transwell migration assays: MOLM13-luc+ cells (A) and MOLM14 cells (B) stimulated with hSDF-1 (100 ng/mL) in the presence of 25 nM NOX-A12, 10 nM midostaurin, or a combination of both. Transwell migration assay incubation times were overnight. These experiments were performed once with multiple cell counts taken for each sample. Data shown are mean +/− S.D.

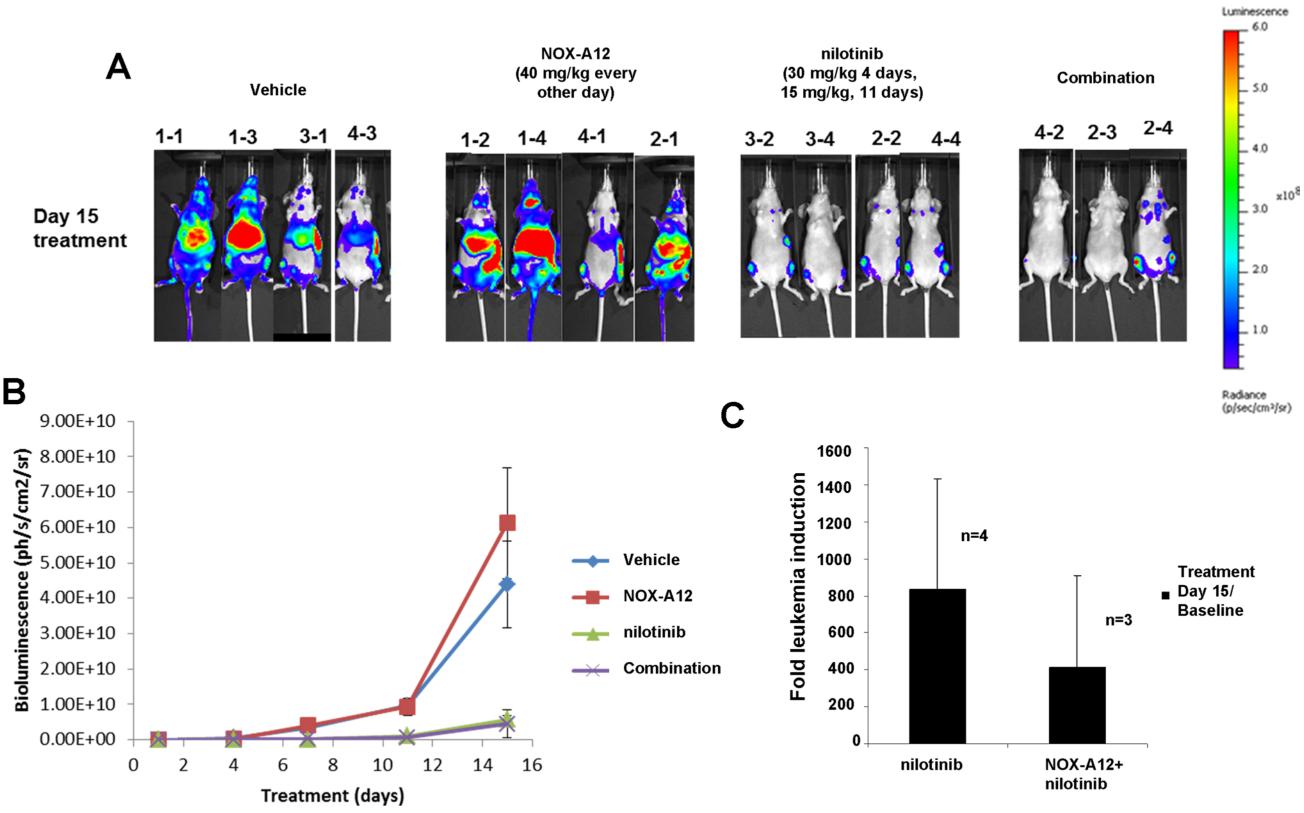

Supplementary Figure 3: NOX-A12 potentiation of effects of ABL inhibition against BCR-ABL-positive cells *in vivo*. 32D.p210 cells were transduced with a retrovirus encoding firefly luciferase (MSCV-Luc), and selected with G418 at a concentration of 1 mg/mL to produce the 32D.p210-luciferase (luc+) cell-line. Bioluminescence imaging was carried out as previously described (Weisberg et al., 2005). Briefly, virus- and *Mycoplasma*-free cells were washed in Hank's Balanced Salt Solution (HBSS; Mediatech, Inc.,VA), resuspended in PBS for injection, and administered via IV tail vein injection (250 mL, 1×10^6 cells) into 15 female NOD.Cg-*Prkdcscid Il2rgtm1Wjl*/SzJ mice (or NOD *scid* gamma (NSG™)) (6 weeks of age) (cat # 005557), purchased from The Jackson Laboratory (Bar Harbor, ME). Mice were imaged 3 days later to establish baseline bioluminescence and were randomized into the following four treatment groups: Vehicles (administered NMP/PEG solution, oral gavage), NOX-A12-only (40 mg/kg sc every other day), nilotinib (30 mg/kg 4 days, oral gavage every day, then 15 mg/kg 11 days, oral gavage, every day), or a combination. Mice were continuously administered treatments until morbidity, at which time mice were preserved in 10% formalin for histopathological analysis. (**A**) Shown are bioluminescence images for mice in the four treatment groups. (**B**) Total bioluminescence values for mice plotted over time. (**C**) Fold induction in leukemia is compared between nilotinib only-treated mice and nilotinib+NOX-A12-treated mice.

1. Weisberg E, Manley PW, Breitenstein W, Brüggen J, Cowan-Jacob SW, Ray A, Huntly B, Fabbro D, Fendrich G, Hall-Meyers E, Kung AL, Mestan J, Daley GQ, et al. Characterization of AMN107, a selective inhibitor of native and mutant Bcr-Abl. Cancer Cell. 2005; 7:129–141.
